# Supplementary material for: A high-resolution mRNA expression time course of embryonic development in zebrafish
Source: eLife. 2017 Nov 16;6:e30860. doi: 10.7554/eLife.30860 (PMC5690287; doi:10.7554/eLife.30860)
Supplement: Supplementary file 6. [file elife-30860-supp6.zip › biolayout-clusters-files/Cluster088.html]

Cluster088


# Cluster088: Detail

### Go to ZFA detail

## GO

| GO ID | Description | Domain | Annotated | Expected | Observed | Adjusted p-value | Genes | Ensembl IDs |
| --- | --- | --- | --- | --- | --- | --- | --- | --- |
| GO:0006355 | regulation of transcription, DNA-templat... | biological\_process | 1072 | 1.49 | 8 | 0.0046 | hoxb6b olig2 nkx2.2a lmx1bb pou3f2a myt1a irx5b irx1a | ENSDARG00000026513 ENSDARG00000040946 ENSDARG00000053298 ENSDARG00000068365 ENSDARG00000070220 ENSDARG00000074030 ENSDARG00000074070 ENSDARG00000101831 |
| GO:0043565 | sequence-specific DNA binding | molecular\_function | 494 | 0.71 | 6 | 0.0084 | hoxb6b nkx2.2a lmx1bb pou3f2a irx5b irx1a | ENSDARG00000026513 ENSDARG00000053298 ENSDARG00000068365 ENSDARG00000070220 ENSDARG00000074070 ENSDARG00000101831 |

  


### Go to GO detail

## ZFA

| ZFA ID | Description | Annotated | Expected | Observed | Fold Enrichment | Adjusted p-value | Genes | Ensembl IDs |
| --- | --- | --- | --- | --- | --- | --- | --- | --- |
| ZFA:0009361 | serotonergic neuron | 11 | 0.01 | 3 | 300 | 0.044 | irx1a lmx1bb nkx2.2a | ENSDARG00000101831 ENSDARG00000068365 ENSDARG00000053298 |
